# Aggressive Mandibular Plasmablastic Lymphoma in an HIV‐Negative Patient: Clinicopathologic and Immunophenotypic Insights From a Rare Oral Case

**DOI:** 10.1155/crid/4470887

**Published:** 2026-07-10

**Authors:** Cláudio Maranhão Pereira, Leonardo Araújo de Andrade, Patrícia Freire Gasparetto, Hidecazio de Oliveira Sousa, Tessa de Lucena Botelho

**Affiliations:** ^1^ Department of Oral Pathology, School of Dentistry, Pontifical Catholic University of Goiás, Goiânia, Brazil, pucgoias.edu.br; ^2^ Department of Oral Surgery, School of Dentistry, Paulista University, Goiânia, Brazil, unesp.br; ^3^ Department of Oral Pathology, School of Dentistry, Alfredo Nasser University Center, Goiânia, Brazil

**Keywords:** B-cell lymphoma, HIV-negative, immunohistochemistry, oral cavity, oral neoplasms, plasmablastic lymphoma

## Abstract

Plasmablastic lymphoma (PBL) is a rare and highly aggressive subtype of diffuse large B‐cell lymphoma characterized by plasmacytic differentiation and a distinct immunophenotypic profile. It is most frequently associated with human immunodeficiency virus (HIV) infection and typically involves the oral cavity. However, PBL may also occur in immunocompetent individuals, representing a diagnostic and clinical challenge. This report describes a rare case of PBL affecting the mandible of an HIV‐negative patient who presented with a rapidly progressive ulcerated oral lesion. Histopathological examination revealed a diffuse proliferation of large atypical cells with plasmablastic morphology and a high mitotic rate. Immunohistochemical analysis demonstrated strong expression of plasma cell markers, including CD138 and MUM1, along with negativity for conventional B‐cell markers such as CD20 and CD79a. The Ki‐67 proliferation index was approximately 90%, confirming the aggressive biological behavior of the neoplasm. Based on these findings, a diagnosis of PBL was established. Despite prompt referral for oncologic management and initiation of chemotherapy, the disease showed rapid progression with an unfavorable outcome. This case highlights the importance of including PBL in the differential diagnosis of aggressive oral lesions, even in HIV‐negative and immunocompetent patients, and emphasizes the critical role of histopathological and immunohistochemical evaluation for accurate diagnosis.

## 1. Introduction

Plasmablastic lymphoma (PBL) is an uncommon and highly aggressive variant of diffuse large B‐cell lymphoma (DLBCL) that typically arises in association with human immunodeficiency virus (HIV) infection or other causes of immunodeficiency, such as posttransplantation or long‐term immunosuppressive therapy [1, 2]. Since its first description by Delecluse et al. [1] in 1997, PBL has been recognized for its distinct clinicopathological features, including plasmacytic differentiation, immunophenotypic profile, and dismal clinical outcome.

The majority of PBL cases occur in HIV‐positive patients, often involving the oral cavity, particularly the gingiva, palate, and jawbones [3, 4]. The oral predilection has been linked to Epstein–Barr virus (EBV) infection, which contributes to oncogenic transformation through latent membrane protein‐1 (LMP‐1) and other viral oncoproteins [5, 6]. Nevertheless, a growing number of PBL cases have been reported in HIV‐negative individuals, suggesting that immune dysregulation alone is not a prerequisite for its development [7–9].

The diagnosis of PBL was historically challenging due to overlapping histopathological features with other hematologic malignancies. However, advances in immunohistochemistry and molecular pathology have significantly improved diagnostic accuracy at the histopathological level. Currently, the main diagnostic challenges are more relevant to the clinical and radiological assessment, as the initial presentation of PBL can closely mimic other aggressive oral lesions, including squamous cell carcinoma, plasmacytoma, and poorly differentiated carcinoma [10, 11]. Immunohistochemistry remains the cornerstone of histopathological diagnosis, as PBL typically expresses plasma cell markers (CD138, MUM1, and CD38) and lacks conventional B‐cell markers (CD20 and PAX5) [12, 13].

Given its rarity in immunocompetent individuals and the clinical diagnostic complexity, reporting such cases is essential to improve understanding of its clinical behavior and therapeutic outcomes. Therefore, the objective of this study is to report a rare case of PBL occurring in an HIV‐negative, immunocompetent patient, highlighting its clinical presentation in the oral cavity, histopathological and immunohistochemical features, and prognostic implications.

## 2. Case Presentation

A 52‐year‐old White male presented to the Oral Medicine Clinic with a 4‐month history of a rapidly enlarging, painless swelling on the left side of the face. The patient reported no significant medical history. Serological testing for HIV performed during the diagnostic workup returned negative, and there was no clinical or laboratory evidence of systemic immunodeficiency.

Clinical examination revealed facial asymmetry associated with a fungating, exophytic ulcerated lesion in the left mandibular region, impairing stomatognathic function (Figure 1). Radiographic examination (orthopantomogram [OPG]) demonstrated extensive osteolytic destruction of the mandibular bone (Figure 2).

**Figure 1:**
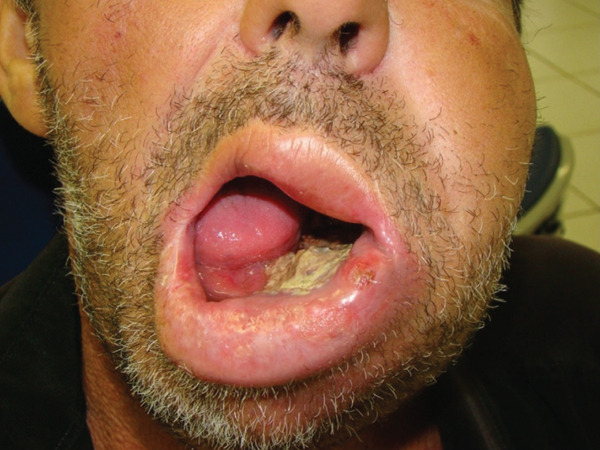
Intraoral aspect showing a fungating, exophytic ulcerated lesion on the left mandibular alveolar ridge, with associated facial asymmetry.

Based on clinical and imaging findings, differential diagnoses included squamous cell carcinoma and non‐Hodgkin lymphoma. An incisional biopsy revealed diffuse proliferation of large atypical plasmablastic cells with a “starry‐sky” pattern (Figure 3).

**Figure 2:**
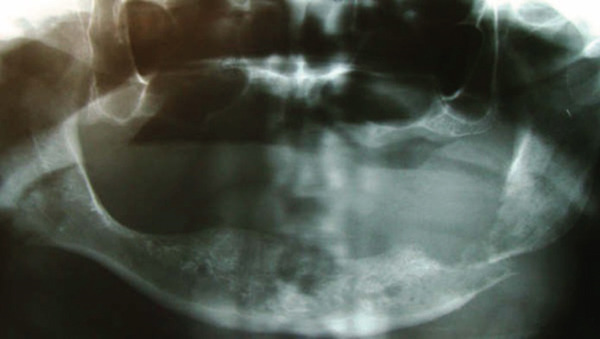
Orthopantomogram (OPG) demonstrating extensive ill‐defined osteolytic destruction of the left mandibular body and ramus, consistent with an aggressive malignant process.

Immunohistochemical analysis demonstrated negative CD20 expression and strong positivity for CD38, CD138, and MUM1 (Figure 4). Tumor cells were negative for CD79a, CD10, CD3, CK, PAX5, and AE1/AE3. The Ki‐67 proliferation index was approximately 90% (Figure 5), confirming the highly aggressive biological behavior of the neoplasm. Based on the histopathological and immunohistochemical findings, a final diagnosis of PBL was established.

**Figure 3:**
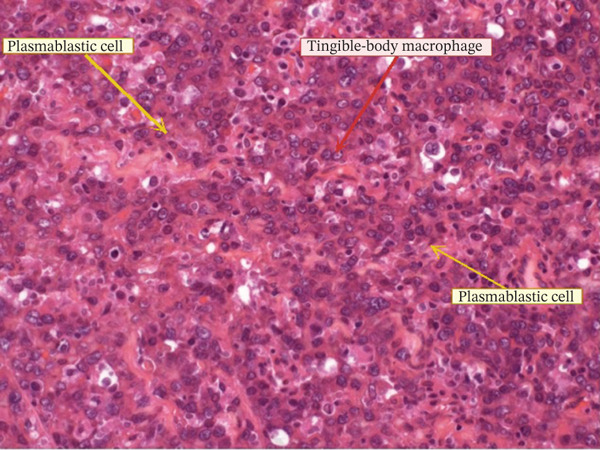
Photomicrograph showing diffuse proliferation of large atypical lymphoid cells with plasmablastic morphology and prominent nucleoli. Note the scattered tingible‐body macrophages creating a “starry‐sky” pattern (H&E, 100x)

**Figure 4:**
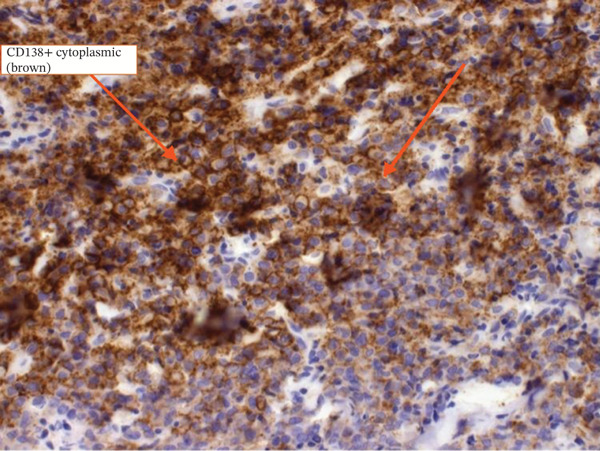
Immunohistochemical staining showing strong cytoplasmic positivity for CD138, confirming plasmacytic differentiation of the neoplastic cells (400x).

**Figure 5:**
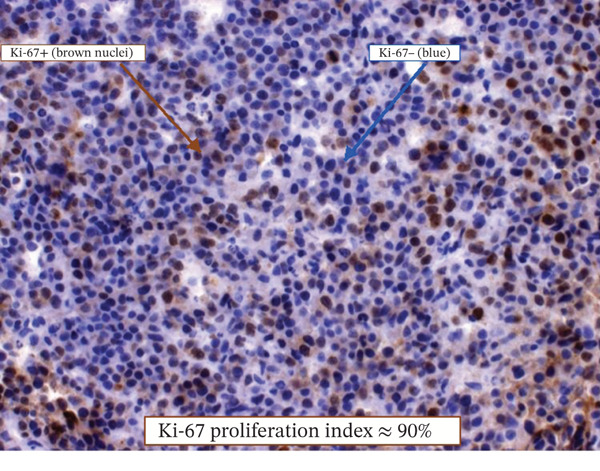
High Ki‐67 labeling index (~90%), consistent with aggressive proliferative behavior (400x).

The patient was referred to an oncology center for staging evaluation and treatment planning. Due to the rapid clinical deterioration at the time of referral, a comprehensive staging workup—including PET‐CT imaging—could not be completed before initiation of therapy. The patient was treated with CHOP (cyclophosphamide, doxorubicin, vincristine, and prednisone) chemotherapy. Although dose‐intensified regimens such as EPOCH or CODOX‐M/IVAC were considered by the oncology team, the patient′s clinical condition and the pace of disease progression precluded their use. Unfortunately, the disease progressed rapidly, and the patient died approximately 50 days after the histopathological diagnosis, shortly after completion of the first CHOP chemotherapy cycle (Figure 6). The immediate cause of death was multiple organ failure secondary to rapid progression of the underlying disease.

**Figure 6:**
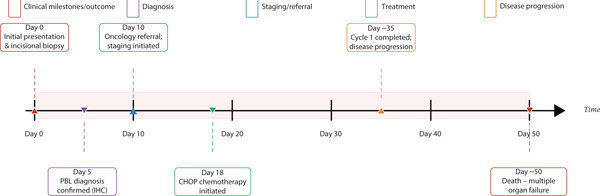
Clinical timeline of the reported case. Day 0 corresponds to the initial clinical presentation and incisional biopsy. The patient received one cycle of CHOP chemotherapy and died approximately 50 days after diagnosis due to multiple organ failure secondary to rapid disease progression.

## 3. Discussion

PBL is a rare and highly aggressive neoplasm originally described in association with HIV infection and predominantly affecting the oral cavity [14, 15]. Although most cases occur in immunocompromised patients, an increasing number of reports have documented PBL in HIV‐negative and immunocompetent individuals, reinforcing that immunodeficiency is not an absolute requirement for its development [16–20]. These cases often present diagnostic challenges due to their rarity and overlapping features with other high‐grade hematolymphoid malignancies.

The clinical presentation observed in the present case—a rapidly progressive fungating ulcerated mandibular lesion in a middle‐aged HIV‐negative male—is consistent with previously reported cases of oral PBL in immunocompetent patients. Morscio et al. [3] noted that HIV‐negative patients with PBL tend to present at older ages and with more varied anatomical sites compared with their HIV‐positive counterparts. The extensive osteolytic destruction documented on the OPG in this case is a radiological feature that, although not exclusive to PBL, has been associated with mandibular involvement in previously published cases and warrants immediate inclusion of hematolymphoid malignancies in the differential diagnosis. In a systematic review of oral PBL, the gingiva was identified as the most frequently involved intraoral site, making mandibular body involvement—as observed here—a less frequent but well‐documented presentation [4]. Compared with other published HIV‐negative cases, the clinical course in this patient was remarkably aggressive: Death occurred within 50 days of diagnosis, representing one of the shortest survival intervals documented in the HIV‐negative PBL subset and reinforcing the dismal prognosis of this entity even in immunocompetent hosts.

The main differential diagnoses of PBL include plasmacytoma, immunoblastic DLBCL, and other poorly differentiated malignant neoplasms. Immunohistochemistry is essential for accurate diagnosis, as PBL typically shows strong expression of plasma cell markers such as CD138 and MUM1, absence of B‐cell markers including CD20 and CD79a, and a markedly elevated proliferation index—features that help distinguish it from plasma cell neoplasms and conventional lymphomas [21–23].

EBV is detected in approximately 70% of PBL cases, predominantly in HIV‐positive patients, suggesting an oncogenic role. However, its presence in HIV‐negative cases is variable and not mandatory for diagnosis [24]. In the present case, EBER in situ hybridization was not performed owing to the unavailability of this technique at the diagnosing institution at the time of the workup. This represents a recognized limitation. Nevertheless, the clinical presentation, histomorphological features, and immunophenotype—including strong CD138 and MUM1 expression, CD20 negativity, and a Ki‐67 index of ~90%—are consistent with previously reported HIV‐negative PBL cases and sufficient to support the diagnosis based on current WHO classification criteria.

Prognosis is generally poor, with a median survival of less than 1 year despite aggressive chemotherapy [25]. Standard regimens such as CHOP are often insufficient, and more intensive protocols such as EPOCH or CODOX‐M/IVAC have been attempted with limited success [26]. In the present case, dose‐intensified therapy was considered but could not be administered due to the patient′s deteriorating performance status at the time of oncological referral, which is consistent with the clinical dilemma frequently encountered in rapidly progressing PBL. Recent studies have explored the use of bortezomib, lenalidomide, and monoclonal antibodies (e.g., daratumumab) with promising results, especially when combined with chemotherapy [27].

Early diagnosis and multidisciplinary management remain crucial. Dentists and oral pathologists play an important role, as oral cavity involvement is often the first manifestation. Biopsy with immunohistochemical analysis is essential to distinguish PBL from other entities such as plasmacytoma, poorly differentiated carcinoma, or melanoma.

A limitation of this case report is the absence of EBV evaluation by EBER in situ hybridization, as discussed above, and the inability to complete a full staging workup prior to the initiation of therapy. These limitations reflect real‐world constraints and are not uncommon in rapidly progressive cases managed in resource‐limited settings. They do not affect the validity of the histopathological diagnosis.

## 4. Conclusion

PBL may occur in HIV‐negative, immunocompetent patients and should be considered in the differential diagnosis of aggressive oral malignancies, particularly in cases presenting with rapidly progressive fungating or ulcerated lesions accompanied by osteolytic bone destruction. Accurate diagnosis relies on careful histopathological evaluation supported by immunohistochemical analysis. Despite advances in therapy, prognosis remains poor, underscoring the importance of early diagnosis and multidisciplinary management.

## Author Contributions

**Cláudio Maranhão Pereira:** conceptualization, clinical diagnosis, writing – original draft, writing – review & editing, supervision. **Leonardo Araújo de Andrade:** surgical procedure, data curation, writing – review & editing. **Patrícia Freire Gasparetto:** data curation, writing – review & editing. **Hidecazio de Oliveira Sousa:** histopathological analysis, writing – review & editing. **Tessa de Lucena Botelho:** histopathological analysis, immunohistochemical analysis, writing – review & editing.

## Funding

No funding was received for this manuscript.

## Disclosure

All authors have read and approved the final version of the manuscript. The corresponding author (Cláudio Maranhão Pereira) had full access to all of the data in this study and takes complete responsibility for the integrity of the data and the accuracy of the data analysis.

## Ethics Statement

This case report was exempt from formal ethics committee review, as it involves a single patient with no experimental intervention. The study was conducted in accordance with the ethical principles of the Declaration of Helsinki. Written informed consent was obtained from the patient′s next of kin prior to publication, including consent for the use of clinical data and accompanying images.

## Consent

Written informed consent was obtained from the patient′s family for publication of this case report and accompanying images.

## Conflicts of Interest

The authors declare no conflicts of interest.

## Data Availability

The data that support the findings of this study are available from the corresponding author upon reasonable request.
